# Automatic infection detection based on electronic medical records

**DOI:** 10.1186/s12859-018-2101-x

**Published:** 2018-04-11

**Authors:** Huaixiao Tou, Lu Yao, Zhongyu Wei, Xiahai Zhuang, Bo Zhang

**Affiliations:** 1 School of Data Science, Fudan University, Shanghai, China; 2 Zhongshan Hospital Affiliated to Fudan University, Shanghai, China

**Keywords:** Electronic medical records, Infection detection, Machine learning, Natural language processing, Automatic disease detection

## Abstract

**Background:**

Making accurate patient care decision, as early as possible, is a constant challenge, especially for physicians in the emergency department. The increasing volumes of electronic medical records (EMRs) open new horizons for automatic diagnosis. In this paper, we propose to use machine learning approaches for automatic infection detection based on EMRs. Five categories of information are utilized for prediction, including personal information, admission note, vital signs, diagnose test results and medical image diagnose.

**Results:**

Experimental results on a newly constructed EMRs dataset from emergency department show that machine learning models can achieve a decent performance for infection detection with area under the receiver operator characteristic curve (AUC) of 0.88. Out of all the five types of information, admission note in text form makes the most contribution with the AUC of 0.87.

**Conclusions:**

This study provides a state-of-the-art EMRs processing system to automatically make medical decisions. It extracts five types of features associated with infection and achieves a decent performance on automatic infection detection based on machine learning models.

## Background

Electronic medical record (EMR) systems have been increasingly and widely adopted in recent years. They were emerging as a rich resource for a variety of research tasks, such as further understandings of genotype-phenotype relationships [1, 2], prediction of antimicrobial resistance [3], as well as the identification of eligible patients for clinical trails [4].

Each EMR is collected in an accumulative way following the clinic diagnostic procedure. It often contains various types of information, including personal information (e.g. age, sex, etc.), narrative admission notes (e.g. past medical history, history of present illness and symptom etc.), vital signs, structured diagnostic test results, medical image diagnoses (e,g. X-ray diagnose etc.), billing codes, discharge notes, and so on. The combination of different data types sets up a barrier for the utilization of EMRs on its secondary usage. Natural language processing (NLP) and machine learning approaches are then introduced to process EMRs and extract valuable information.

A promising line of research based on EMRs are *automatic disease detection* [5–7], which shows great potential to support clinical decision making. Recent studies have shown that appropriate disease algorithms constructed on top of EMRs can contribute to the accurate detection of a wide range of diseases [8], including the identification of subjects with polycystic ovary syndrome [9] and the prediction of the risk of coronary artery disease [10] and so on.

Some existing researches used a single type of data to identify diseases. Hospital discharge summaries were used to predict early psychiatric readmission [11]; narrative portion of emergency department records were leveraged to detect influenza [12]; structured disease classification codes were used to identify individuals in need of testing for celiac disease [13]. On the other hand, researchers also explored to develop algorithms on top of multiple types of data in EMRs for different disease identification, e.g. dementia [14], diabetes [15], depression [16] and rheumatoid arthritis [17–19]. Experimental results showed that combining multiple types of data from EMRs can improve the performance of automatic disease detection [7, 20].

To date, few studies focused on automatic detecting infection using EMRs, which is the most common condition in emergency surgery department. Infection, the invasion of host organism by microorganism, includes acute abdominal diseases, superficial infection, and abscess. With the treatment delayed, infection may advance to sepsis and septic shock, which are life-threatening [21]. Besides, some diagnosis can not be confirmed until microbial culture, which takes long time. Therefore, the identification of infection before the symptoms worsen is crucial.

In this paper, we focus on detecting infected patient before being hospitalized. We collected 8,642 unique patient records from emergency department at Zhongshan Hospital between year 2012 and 2016. After digging deep into the dataset, we found that more than 43% of patient suffered from infection before being hospitalized. There are two main contributions in this work. First, we analyzed the EMRs to identify the distribution of patients with different factors (disease and age). Then we automatically extracted some infection related features from EMRs. “Shifting pain in right quadra” and “Malignant tumor(MT)” are highly relevant to infection. Based on these extracted features, we proposed a method for automatic disease diagnosis, to help doctors with infection identification. Experimental results demonstrated that combining multiple EMR data types of features has the best predictive effects. The method proposed can also be generalized to predict disease gene [22, 23], calculate biomedical ontology-based similarity [24, 25] and identify phenotype similarity [26–28].

## Method

### Dataset collection

**Fig. 1:**
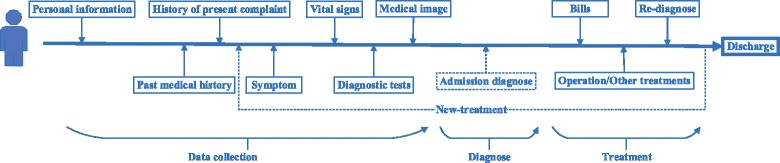
The diagnostic process in clinic

Each piece of EMR is collected in an accumulative way following a diagnostic procedure in clinic as shown in Fig. 1. Corresponding clinical results and medical notes are recorded to form the medical record along this procedure.

#### Personal information

Initially, patients are asked to complete some fundamental information (from other people close to the patient as well), such as age, sex, address etc.

#### Admission note

Second, patients are required to provide information about the past medical history, history of present illness and their unusual states in present, also known as symptom. Doctors may discover some additional symptoms.

#### Diagnose tests and vital signs

Then, patients take various diagnostic tests. A diagnostic test is a medical test performed to assist the diagnosis. Diagnostic tests can also be used to provide prognostic information on people with established disease. In this stage, vital signs (e.g. blood pressure and pulse) are monitored as well.

#### Medical image

Before doctors can make a certain diagnosis, further medical tests, such as medical imaging, will be performed or scheduled to provide more support information. Diagnosis result for the medical image will be recorded.

#### Admission diagnose, bills and treatments

The records and test results above help doctors in admission diagnose, which plays an important role in consequent operations and treatments. Based on the admission diagnose, doctors make initial treatment decisions (e.g. bills and operation).

#### Re-diagnose and new-treatment

If a patient exhibits any unexpected symptoms during the treatment, the above processes will be repeated to make a new diagnosis and to update treatment correspondingly.

Along this procedure, personal information, vital signs, diagnose test results and billing codes are recorded as structured data. Admission note, medical image diagnosis result, admission diagnoses, bills and treatments are recorded as narrative text.

In this paper, we focus on detecting infected patients before they are hospitalized. Therefore, we treat admission diagnose generated by a doctor as the ground-truth information. Personal information, admission notes, diagnostic test results, vital signs and medical image diagnosis are utilized for automatic infection detection.

EMRs used in this paper were collected by the surgical emergency department in Zhongshan Hospital, Fudan University. Ethical approval in this study was obtained from the ethics committee of Zhongshan Hospital. Before we obtained the dataset, all the records were anonymized. Privacy information of patient (name, address and ID) and doctor (name) was removed. In the meantime, the cure ID and admission ID were transformed into random hash numbers, which were used as the unique identification for a specific patient. We further removed incomplete records and this resulted in a dataset with 8,642 records in total.

### Annotations

Although the diagnosis result for infection is recorded in admission diagnoses, there is no direct label available. We thus need to analyze the admission diagnosis text to obtain the ground-truth label. To avoid the huge labor-effort for manual annotation, we propose an automatic way for infection annotation. A list of infection-related terms (see Table 1) were constructed by an expert. Based on the list, 3797 records were identified as infected cases by key word matching on admission diagnoses. To evaluate the performance of the automatic annotation approach, we randomly sample 200 records and ask for manual annotations from domain experts. Table 2 shows that the PPV of automatic annotation is 83%, and the F1-score is close to 84%, which proves the effectiveness of our automatic annotation approach (more information about the evaluation metrics will be given later).

**Table 1:** The diagnostic term defined as infection

	Infection
	Abscess
	Necrosis
	Gangrene
	Pyogenic
	Sepsis
	Erysipelatous
	Pneumonia
	Pyothorax
	Mastitis
	Perforation
	Peritonitis
	Acute cholecystitis
	Gangrenous cholecystitis
	Acute attacking of chronic cholecystitis
	Acute cholangitis
	Acute suppurative cholangitis
	Acute gangrenous cholangitis
	Biliary pancreatitis
	Acute appendicitis
	Acute suppurated appendicitis
	Acute gangrened appendicitis
	Acute purulent gangrenous appendicitis
	Acute phlegmonous appendicitis
	Systemic inflammatory response syndrome
	Sepsis
	Septic shock
	Acute attacking of chronic appendicitis

**Table 2:** Performance of automatic annotation approach compared with manual annotation

Category	PPV	Sensitivity	F1-score
No infection	0.84	0.83	0.84
Infection	0.83	0.84	0.83
Avg / Total	0.84	0.83	0.84

### Basic analysis of the EMRs

**Fig. 2:**
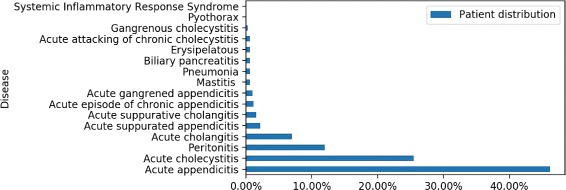
The distribution of patients for different infectious diseases in the EMRs

**Fig. 3:**
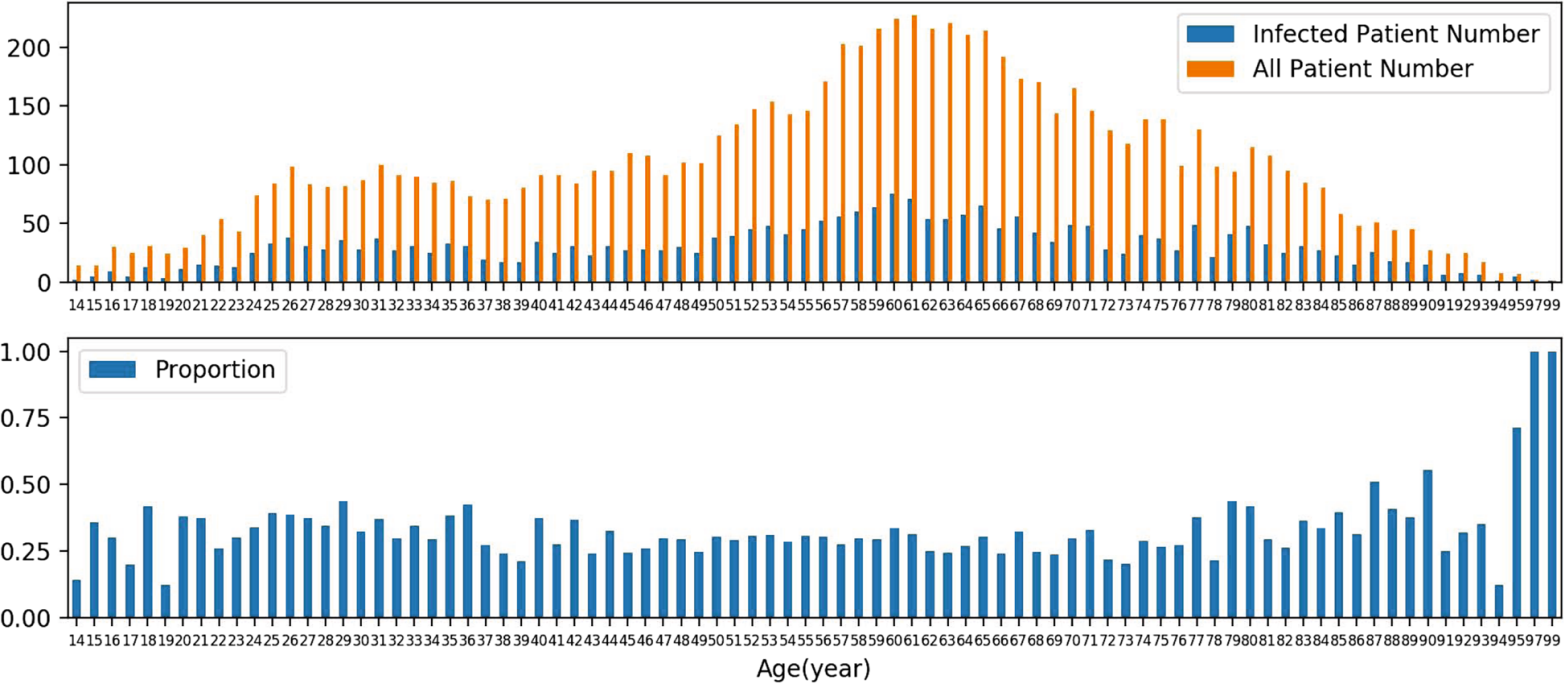
The distribution of patients in terms of ages by number (above) and by proportion (below)

In the EMRs dataset used in this study, nearly 44% of patients suffered from infectious diseases. A total of 16 types of infectious diseases were found by mapping infection-related terms to infectious diseases. The distribution of patients for different infectious diseases can be seen in Fig. 2. It shows that more than 40% of infected patients suffered from acute appendicitis and nearly 25% of infected patients suffered from acute cholecystitis. The number and percentage of infected patients in terms of the age can be found in Fig. 3. There is no apparent clue that patients in some specific age group bear higher risk for infection. The average proportion of infected patients in all patients is 33%.

### Feature development

**Fig. 4:**
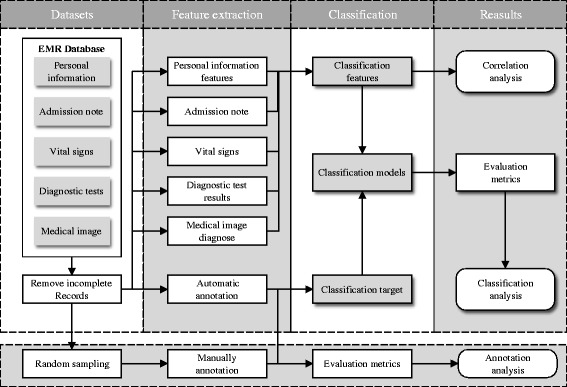
The pipeline for preprocessing and feature extraction on EMRs

The overall framework for EMRs processing and disease identification can be seen in Fig. 4. In order to explore the effectiveness of different types of data for infection identification, we split each record into five parts: personal information, admission note, medical image diagnose, diagnostic test results and vital signs. Note that admission note and medical image diagnose in each medical record are in text form, while personal information, vital signs and diagnostic tests are numerical values.

#### Feature extraction

**Fig. 5:**
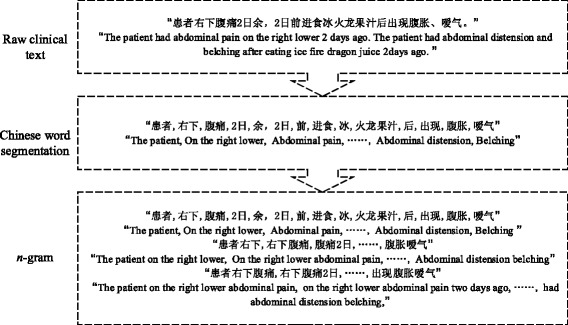
Example for EMR narratives tokenization and n-gram generation

*Admission note* basically consists of narrative past medical history, history of present illness and symptoms. Patients’ past medical history contains family medical history, history of allergy, the diseases the patient has suffered with corresponding treatments, and so on. *Symptoms* are abnormal conditions observed by patients or doctors. Some example symptoms are lower abdominal pain, vomiting, etc. *Medical image diagnoses* are formed of one or two descriptive sentences written by experts for the medical images, such as X-ray, magnetic resonance imaging (MRI) and so on. To process narrative texts (Fig. 5), we utilized some tools from natural language processing. Text segmentation was performed using FNLP [29]. We imported 22 medical thesaurus from *Sogou* [30] to expand the original dictionary. N-grams were extracted, including unigram, bigram and tri-gram. Binary features were used: either present or absent in a patient record.

For *Personal information*, we kept age, sex, medical insurance type, cost and urgency degree. *Vital signs* consist of blood pressure, pulse (heart rate), urine and breathing rate (respiratory rate). We converted the vital signs to numerical features using rule-based approaches (rules were constructed by experts). *Diagnose tests* include the count of white blood cell, total hemoglobin, basophils and body temperature etc. For missing values, we filled the slot by the mean of values of that category.

#### Feature filtering

The feature-level filtering aims to remove sparse and unrelated features. In general, features with lower occurrences in the dataset bring noisy to classification model. We thus used a threshold based approach for feature filtering. All the thresholds were set empirically. In practice, n-gram features extracted from narrative text were filtered out if they appeared less than 500 times. Features extracted from numerical data were filtered out if they appear less than 100 times. We obtained 6 features from personal information, 2276 features from narrative admission notes, 14 features from vital signs, 175 features from diagnose test and 19 features from medical image diagnose. The detail information can be seen in Table 3. Other alternative approaches for feature selection can be explored to further improve the performance, we will leave this for our future work.

**Table 3:** Distribution of features in terms of data types

Feature category	Number	Type
Personal information	6	Number
Admission note	2276	Text
Vital signs	14	Number
Diagnostic tests	175	Number
Medical image diagnose	19	Text
Total	2490	Number/Text

### Experiment setup

#### Model comparison

We compared four different machine learning approaches for infection detection, including logistic regression (LR), naive bayes (NB), gradient boosting classifier (GBC) and random forest (RF). We used *sklearn* [31] for model implementation. To explore the effectiveness of features of various data types, we tested different combinations of features: I) personal information only, II) admission note only, III) vital signs only, IV) diagnose test results only, V) medical image diagnoses only, I&III) personal information and vital signs, I&IV) personal information and diagnose test results, II&V) text-based features (combination of features from admission note and medical image diagnoses), II&IV&V) admission note, diagnose tests and medical image diagnoses, I&II&V) personal information and features from admission note and medical image diagnoses, I&II&IV&V) personal information and features from admission note and medical image diagnoses, I&II&II&IV&V) total (combination of all five type of features).

#### Evaluation metrics

To evaluate the effectiveness of different models, we used micro-averaged positive predictive value (PPV), sensitivity, area under the receiver operating characteristic curve (AUC) and f1-score as evaluation metrics. PPV and sensitivity are defined as follows:

1 $$\begin{array}{*{20}l} PPV&= \frac{{TP}}{{TP}+{FP}} \end{array} $$

2 $$\begin{array}{*{20}l} Sensitivity&= \frac{{TP}}{{TP}+{FN}} \end{array} $$

where *TP*, *FP* and *FN* represent true positives (correctly predicted infection), false positives (incorrect predicted infection) and false negatives (correct predicted un-infection). F1-score is the comprehensive measurement of PPV and sensitivity with equal weights according to the following formula:

3 $$ F1=2\times \frac{PPV\times Sensitivity}{PPV+Sensitivity}  $$

## Results

### Correlation between single feature and infection

Exploratory analysis of correlation between features and infection were provided in terms of both linear and non-linear metrics. The linear correlation is measured by Pearson correlation coefficient. The non-linear correlation is carried out through mutual information coefficient (MIC). The top six features sorted by Pearson correlation coefficient were *shifting pain in right quadrant*, *malignant tumor(MT)*, *mcburney point*, *fluctuation*, *white blood cell count(WBC)*, *age* and *lymphocytes percentage(LYMPH%)*. The details are shown in Table 4. Acute appendicitis is an infectious disease, whose main manifestations include shifting pain in right quadrant and a fixed tenderness on mcburney point. Besides, patients with malignant tumor(MT) always have poor immunity. Some gastrointestinal tumors may result in ileus and then easily progress to acute peritonitis. The abscess on body surface, a kind of infection, would presents a fluctuation. Once patients get infected, white blood cell, as an inflammatory marker, will increase and meanwhile the lymphocytes percentage correspondingly correspondingly decreases. This features prove the effectiveness of the feature extraction process, and presents a useful guideline for future EMR studies.

**Table 4:** The top six features associated with infection

Features	EMR components	Correlation coefficient(*p*<0.001)	MIC
Shifting pain in right quadrant	Admission note	0.32	0.09
Malignant tumor(MT)	Admission note	0.32	0.09
Mcburney point	Admission note	0.23	0.04
Fluctuation	Admission note	0.22	0.04
White blood cell count(WBC)	Diagnose test	0.20	0.03
Lymphocytes percentage(LYMPH%)	Diagnose test	-0.12	0.04

### Performance of different feature categories

We split 8642 patient records into training and test set (80% vs 20%). In order to provide a reliable performance evaluation, we conduct 5-fold cross validation. The results of different feature categories with different models are shown in Table 5. The best prediction results (0.88) are generated by gradient boosting classifier (GBC) using all features (combination of all five type of features). Among the five single components, admission note generates the best performance with the area under curve (AUC) score of 0.87. The performance of all models using single feature of diagnose test results, personal information or vital signs are poor (0.51-0.66) in terms of both f1-score and AUC. However, by combining personal information and vital signs, performance can be improved to a range between 0.68 to 0.72. The AUC of using text-based features (combination of admission note and medical image diagnose) scores 0.87, which is quite close to the best performance using all features.

**Table 5:** The results of different feature categories with different models

Model	Feature type	AUC	F1-score	PPV	Sensitivity
Random Forest	Personal information (I)	0.62	0.56	0.61	0.52
	Admission notes (II)	0.83	0.81	0.86	0.77
	Vital signs (III)	0.61	0.55	0.61	0.5
	Diagnose tests (IV)	0.51	0.07	0.56	0.04
	Medical image diagnoses(V)	0.51	0.06	0.76	0.03
	I & III	0.68	0.63	0.68	0.59
	I & IV	0.63	0.57	0.61	0.53
	II & V	0.84	0.81	0.86	0.77
	II & IV & V	0.83	0.81	0.86	0.77
	I & II & V	0.83	0.81	0.86	0.77
	I & II & IV & V	0.84	0.82	0.86	0.78
	Total (I & II & III & IV & V)	0.84	0.82	0.86	0.79
Logistic Regression CV	Personal information (I)	0.67	0.62	0.65	0.59
	Admission note (II)	0.87	0.85	0.85	0.86
	Vital signs (III)	0.59	0.48	0.6	0.4
	Diagnose test (IV)	0.51	0.09	0.54	0.05
	Medical image (V)	0.51	0.06	0.8	0.03
	I & III	0.68	0.65	0.66	0.64
	I & IV	0.68	0.65	0.65	0.65
	II & V	0.87	0.85	0.85	0.86
	II & IV & V	0.87	0.86	0.85	0.87
	I & II & V	0.87	0.85	0.85	0.86
	I & II & IV & V	0.87	0.85	0.85	0.86
	Total (I & II & III & IV & V)	0.87	0.86	0.86	0.87
Bernoulli NB	Personal information (I)	0.58	0.58	0.53	0.65
	Admission note (II)	0.65	0.69	0.55	0.93
	Vital signs (III)	0.6	0.52	0.59	0.46
	Diagnose test (IV)	0.55	0.63	0.48	0.9
	Medical image (V)	0.51	0.06	0.71	0.03
	I & III	0.6	0.52	0.59	0.46
	I & IV	0.55	0.63	0.48	0.9
	II & V	0.66	0.7	0.56	0.93
	II & IV & V	0.67	0.71	0.57	0.93
	I & II & V	0.66	0.7	0.56	0.93
	I & II & IV & V	0.67	0.71	0.57	0.93
	Total (I & II & III & IV & V)	0.68	0.71	0.58	0.93
Gradient Boosting Classifier	Personal information (I)	0.66	0.62	0.66	0.58
	Admission note (2)	0.87	0.85	0.85	0.86
	Vital signs (III)	0.65	0.6	0.63	0.58
	Diagnose test (IV)	0.51	0.09	0.59	0.05
	Medical image (V)	0.51	0.06	0.78	0.03
	I & III	0.72	0.69	0.69	0.69
	I & IV	0.67	0.63	0.65	0.62
	II & V	0.87	0.85	0.85	0.86
	II & IV & V	0.87	0.86	0.85	0.87
	I & II & V	0.87	0.86	0.86	0.86
	I & II & IV & V	0.87	0.86	0.86	0.87
	Total (I & II & III & IV & V)	0.88	0.86	0.86	0.87

## Discussion

In this work, we demonstrated a feature extraction procedure for EMRs. This procedure generated five types of features for infection detection, which presented a guideline to future infection related study. Second, we used machine learning methods to detect infected patients automatically. Experimental results showed that the proposed method achieved promising predictive ability, which can be used to help doctors with infection identification. There are several limitations. Due to the lack of Chinese medical resource like Unified Medical Language System (UMLS), we can only extract feature features extracted via a manually constructed word list. This can only cover part of target medical concept which limits the performance of our system. Second, medical tests taken are in-consistent across patients and this results in a sparse problem for features extracted from diagnostic tests. This hurts the performance of our system.

## Conclusion

This study provides a state-of-the-art EMRs processing system to automatically make medical decision. The single factor correlation analysis shows that the processing system is able to identify indicative factors for the detection of infection. We also analyze the effectiveness of different types of features for infection detection and reveal the effectiveness of text-based features. The system, using all features achieves the best performance with AUC over 88%. In future, we will explore to use reinforcement learning based approach [32, 33] to collect diagnosis information automatically.
